# Metabolic Aspects of Adenosine Functions in the Brain

**DOI:** 10.3389/fphar.2021.672182

**Published:** 2021-05-14

**Authors:** Mercedes Garcia-Gil, Marcella Camici, Simone Allegrini, Rossana Pesi, Maria Grazia Tozzi

**Affiliations:** ^1^ Department of Biology, Unit of Physiology, University of Pisa, Pisa, Italy; ^2^ Interdepartmental Research Center “Nutraceuticals and Food for Health”, University of Pisa, Pisa, Italy; ^3^ Department of Biology, Unit of Biochemistry, University of Pisa, Pisa, Italy

**Keywords:** adenosine, 5′-nucleotidases, adenosine deaminase, adenosine kinase, S-adenosylhomocysteine hydrolase, nucleoside transporters, brain, metabolism

## Abstract

Adenosine, acting both through G-protein coupled adenosine receptors and intracellularly, plays a complex role in multiple physiological and pathophysiological processes by modulating neuronal plasticity, astrocytic activity, learning and memory, motor function, feeding, control of sleep and aging. Adenosine is involved in stroke, epilepsy and neurodegenerative pathologies. Extracellular concentration of adenosine in the brain is tightly regulated. Adenosine may be generated intracellularly in the central nervous system from degradation of AMP or from the hydrolysis of S-adenosyl homocysteine, and then exit via bi-directional nucleoside transporters, or extracellularly by the metabolism of released nucleotides. Inactivation of extracellular adenosine occurs by transport into neurons or neighboring cells, followed by either phosphorylation to AMP by adenosine kinase or deamination to inosine by adenosine deaminase. Modulation of the nucleoside transporters or of the enzymatic activities involved in the metabolism of adenosine, by affecting the levels of this nucleoside and the activity of adenosine receptors, could have a role in the onset or the development of central nervous system disorders, and can also be target of drugs for their treatment. In this review, we focus on the contribution of 5′-nucleotidases, adenosine kinase, adenosine deaminase, AMP deaminase, AMP-activated protein kinase and nucleoside transporters in epilepsy, cognition, and neurodegenerative diseases with a particular attention on amyotrophic lateral sclerosis and Huntington’s disease. We include several examples of the involvement of components of the adenosine metabolism in learning and of the possible use of modulators of enzymes involved in adenosine metabolism or nucleoside transporters in the amelioration of cognition deficits.

## Introduction

Adenosine regulates multiple physiological and pathophysiological processes, by acting both through G-protein coupled adenosine receptors and intracellularly. It modulates neuronal plasticity (Sebastiao and Ribeiro, 2015), astrocytic activity (Agostinho et al., 2020), learning and memory (Chen, 2014; Simoes et al., 2016; Bannon et al., 2017; Perrier et al., 2019; Temido-Ferreira et al., 2019; Zhang et al., 2020), food intake (Kola, 2008), motor function (Mori, 2020), sleep/wake cycle (Donlea et al., 2017; Lazarus et al., 2019), pain (Vincenzi et al., 2020), immunosupression (Vijayan et al., 2017), proliferation (Jacobson et al., 2019), and aging (Costenla et al., 2011). Adenosine is involved in ischemia and stroke (Williams-Karnesky and Stenzel-Poore, 2009; Melani et al., 2014; Pereira-Figueiredo et al., 2021), epilepsy (Boison and Jarvis, 2020; Tescarollo et al., 2020), and neurodegenerative pathologies such as Parkinson’s disease (PD) (Fredholm and Svenningsson, 2020; Glaser et al., 2020), Alzheimer’s disease (AD) (Rahman, 2009; Cunha and Agostinho, 2010; Cellai et al., 2018), amyotrophic lateral sclerosis (ALS) (Ng et al., 2015; Sebastiao et al., 2018), and Huntington’s disease (HD) (Lee and Chern, 2014). Extracellular adenosine, interacting with P1 receptors (A1R, A2AR, A2BR, and A3R) regulates metabolism through different signaling pathways. In fact, the binding of adenosine to A1R and A3R activates the G_i/o_ family of G-proteins, which inhibits adenylate cyclase, while the binding to A2AR and A2BR, through activation of G_s_ protein, stimulates the production of cyclic AMP (Burnstock, 2017), thus contributing to the fine-tuning of synapses and to the coordination of neuronal circuitry (Agostinho et al., 2020). In neurons and astrocytes, heteromers can be formed by interaction of A1R with A2AR, A2AR with A2BR, and A2AR with dopamine D2, D3 and D4 receptors (Guidolin et al., 2020). The interactions between receptors in these complexes modify the signaling and function of the individual receptors and lead to signal integration in the central nervous system. For example, the heteromer A2AR-D2 receptor is found in the striatum where it transforms the dopamine-facilitated inhibition into adenosine-induced activation of adenylate cyclase (Ferré and Ciruela, 2019). Higher order heteroreceptor complexes of A2AR-D2 receptor with either-metabotropic glutamate receptor 5 or Sigma1 receptor have also been described (Borroto-Escuela et al., 2020). It has been hypothesized that heteroreceptors could be involved in learning and memory, including memory associated drug addition and that heteroreceptor complexes could be a target for the treatment of schizophrenia, drug addition, and PD (Borroto-Escuela et al., 2020, 2021; Glaser et al., 2020).

**FIGURE 1:**
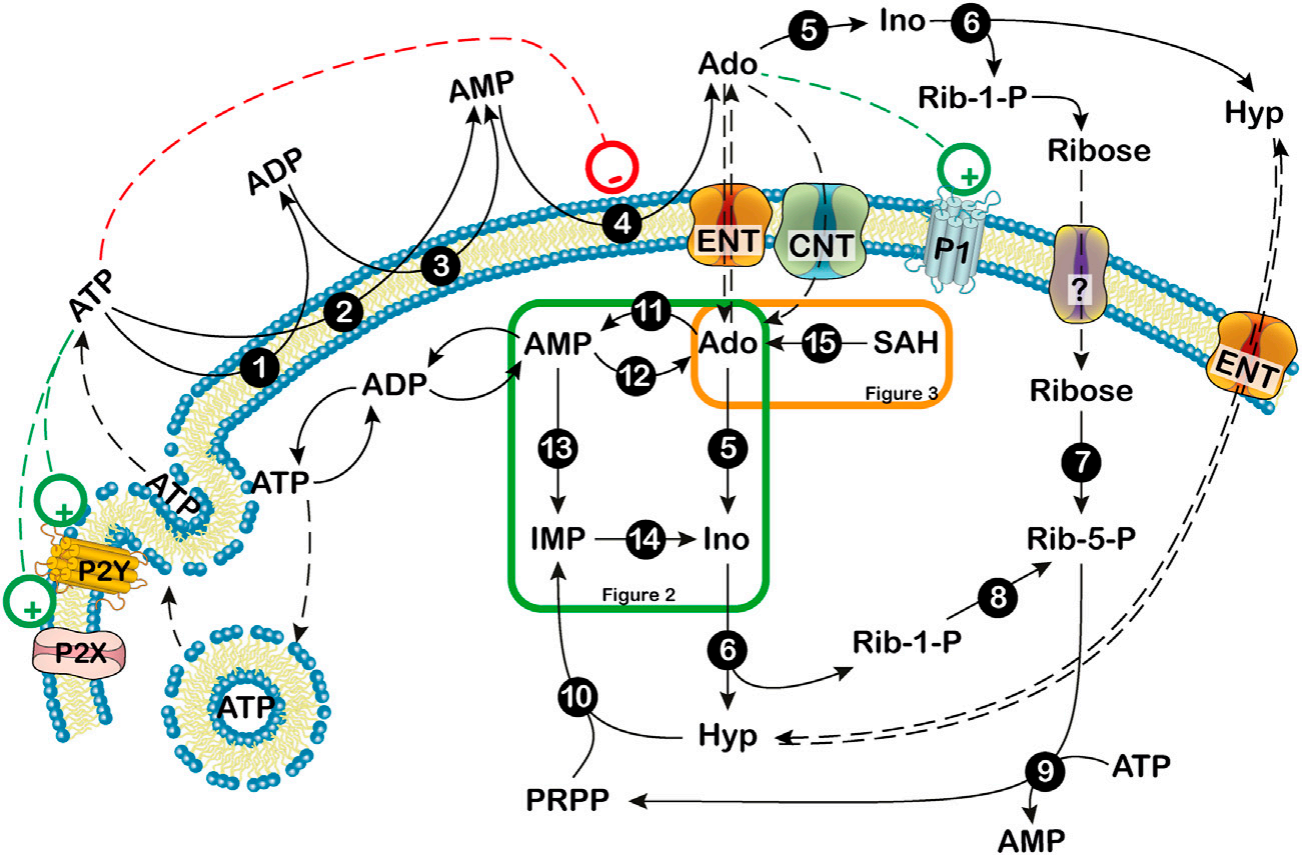
Extra- and intracellular adenosine production. Extracellularly, ATP can be dephosphorylated to AMP by ectonucleoside triphosphate diphospho-hydrolase (CD39) or ecto-nucleotide pyrophosphatase/phosphodiesterase. Then, AMP can be dephosphorylated to adenosine by the extracellular 5′-nucleotidase, CD73. Extracellular adenosine can be converted into hypoxanthine (Hyp) and ribose-1 phosphate (Rib-1-P) by the combined action of ectosolic adenosine deaminase and purine nucleoside phosphorylase. Extracellular Rib-1-P might be dephosphorylated by membrane phosphatases and equilibrates with the intracellular ribose through a not yet defined transporter (?). Inside the cell, at low energy charge, adenosine originates mainly from AMP and can be exported or deaminated. When extracellular adenosine generated from ATP breakdown is transported inside the cell, it might be phosphorylated by the low K_M_ ADK or deaminated by the high K_M_ ADA if adenosine reaches high levels. 1,3: ecto-nucleoside triphosphate diphosphohydrolase; 2: ecto-nucleotide pyrophosphatase/phosphodiesterase; 4: ecto-5′-nucleotidase; 5: adenosine deaminase; 6: purine nucleoside phosphorylase; 7: ribokinase; 8: phosphoribomutase; 9: 5-phosphoribosyl-1-pyrophosphate synthetase; 10: hypoxanthine guanine phosphoribosyltransferase; 11: adenosine kinase; 12: cytosolic 5′ nucleotidase I; 13: AMP deaminase; 14: cytosolic 5′ nucleotidase II; 15: S-adenosylhomocysteine hydrolase. Ado: adenosine; CNT: concentrative nucleoside transporter; ENT: equilibrative nucleoside transporter; Hyp: hypoxanthine; Ino: inosine; P1: purinergic receptor type 1; P2: purinerigic receptor type 2; Rib-1-P: ribose-1-phosphate; Rib-5-P: ribose-5-phosphate. Green and orange boxes indicate that these pathways are described in more details in Figures 2, 3. +: stimulation; -: inhibition.

**FIGURE 2:**
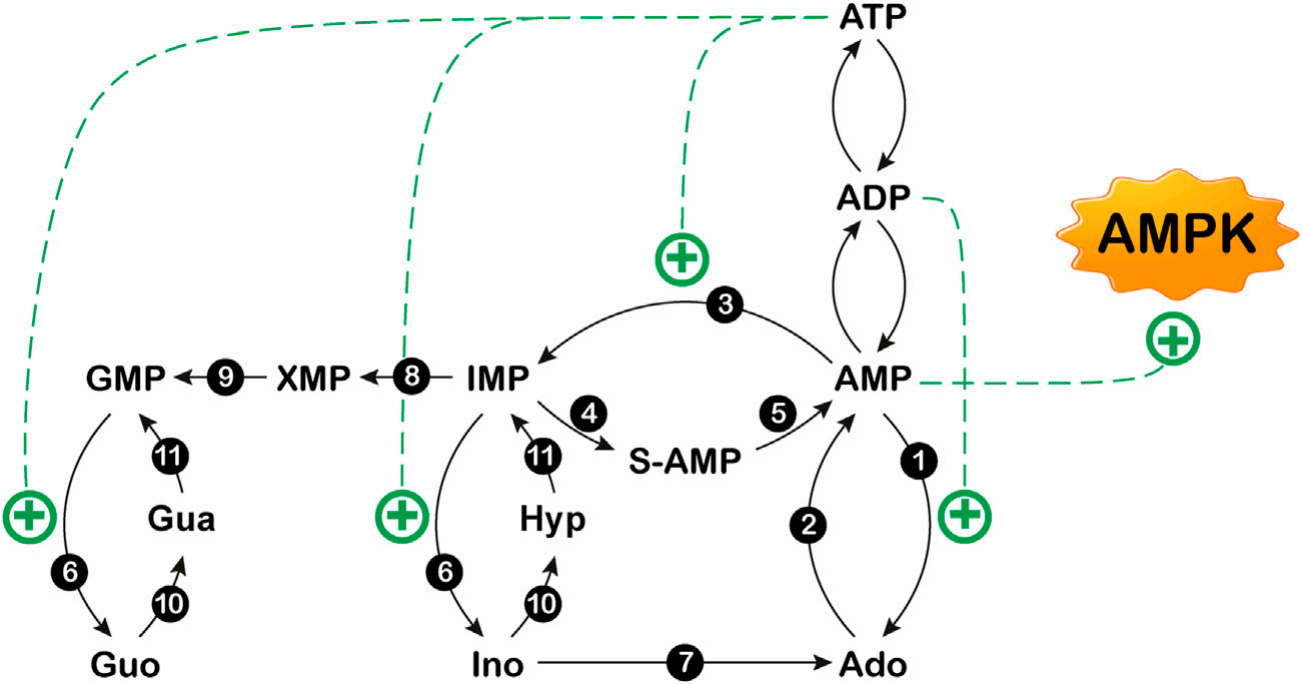
Purine nucleotide cycles. 1: 5′-nucleotidase I; 2: adenosine kinase; 3: AMP deaminase; 4: adenylosuccinate synthase; 5: adenylosuccinate lyase; 6: cytosolic 5′-nucleotidase II; 7: adenosine deaminase; 8: IMP dehydrogenase; 9: GMP synthase; 10: purine nucleoside phosphorylase; 11: hypoxanthine guanine phosphoribosyltransferase. The figure also shows that AMP is an activator of AMP-activated protein kinase (AMPK). Ado: adenosine; Gua: guanine; Guo: guanosine; Hyp: hypoxhanthine; Ino: inosine. S-AMP: succinylAMP. +: stimulation.

**FIGURE 3:**
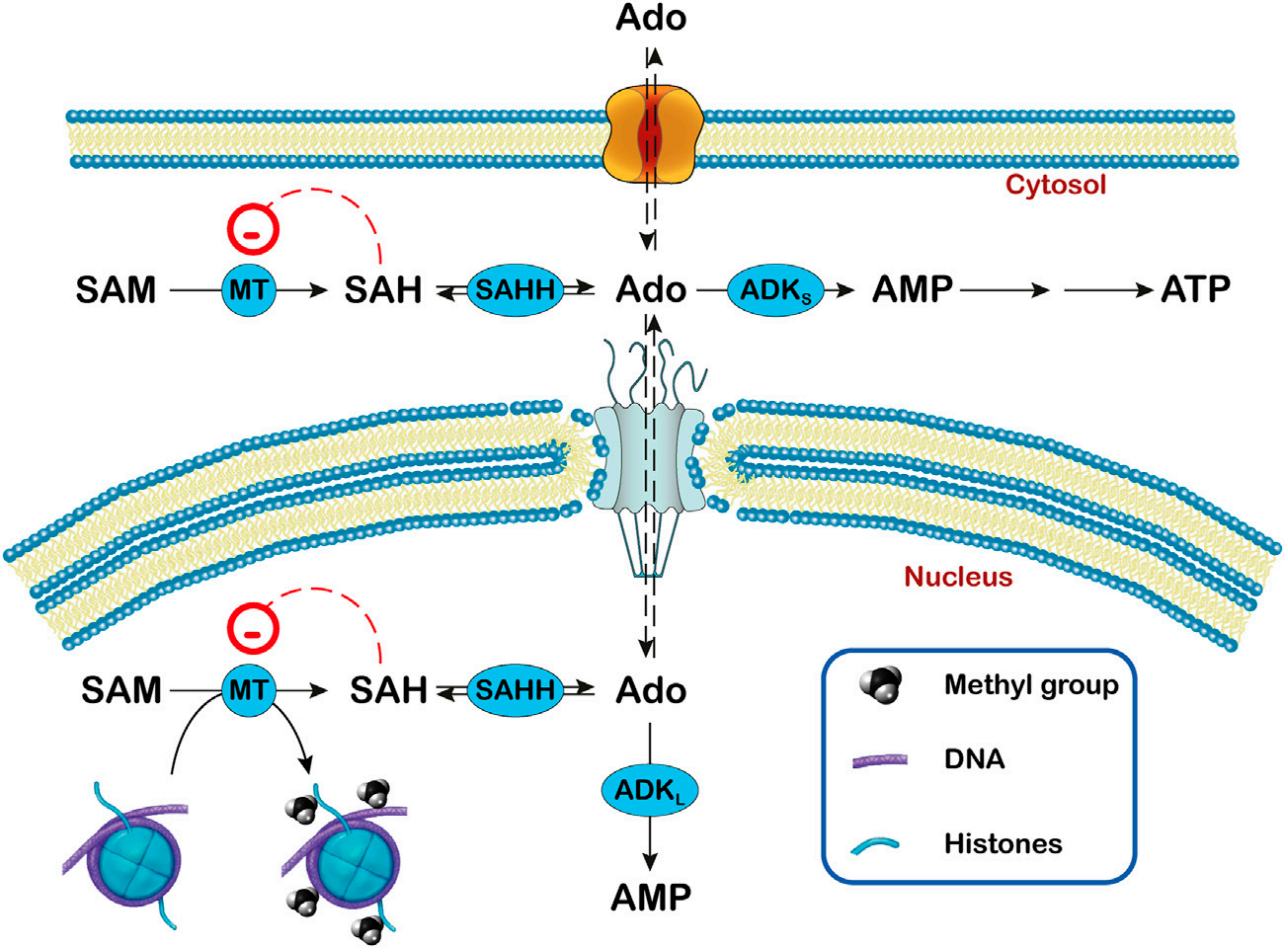
Relationship between adenosine, ADK and transmethylation reactions in subcellular compartments. In the transmethylation reactions catalyzed by methyltransferases (MT), S-adenosylmethionine (SAM) donates the methyl group to various acceptors and is converted to S-adenosylhomocysteine (SAH), which generates adenosine (Ado) by the action of S-adenosylhomocysteine hydrolase (SAHH). In the nucleus, the methyl group can be transferred to DNA and histones. The activity of ADK (ADK_S_ in the cytoplasm and ADK_L_ in the nucleus) decreases the concentration of Ado and favors the transmethylation reactions. In this way, ADK_L_ contributes to the DNA methylation in the nucleus.

The concentration of extracellular adenosine depends on both intracellular and extracellular purine nucleotide catabolism and on the traffic of the nucleoside across the membrane through specific transporters (Baldwin et al., 2004; Pastor-Anglada and Pérez-Torras, 2018; Camici et al., 2018b). Extracellular adenosine can arise from the degradation of intracellular ATP (Figure 1) (Camici et al., 2018b). At high energy charge, mainly inosine and hypoxanthine are generated by ATP catabolism. In fact, in these conditions, both the cytosolic 5’-nucleotidase II (NT5C2) and AMP deaminase (AMPD) are allosterically activated by ATP (Figure 2) (Ashby and Holmsen, 1983; Tozzi et al., 2013), therefore AMP is deaminated by AMPD into IMP, which is dephosphorylated into inosine by NT5C2. Conversely, at low energy charge, the accumulation of AMP inside the cell, leads to the activation of a specific AMP-activated protein kinase (AMPK), the main regulator of cellular energy homeostasis (Hardie et al., 2012; Rosso et al., 2016; Peixoto et al., 2017; Liu et al., 2020) (Figure 2). The dephosphorylation of AMP by a high K_M_ AMP-specific 5’-nucleotidase I (NT5C1) which is strongly activated by ADP, leads to increased intracellular adenosine (Skladanowski and Newby, 1990). In such conditions, adenosine might be deaminated, but the K_M_ of adenosine deaminase (ADA) for adenosine is sufficiently high (25–150 µM) (Ford et al., 2000; Ipata et al., 2011) to favor adenosine increase and exit through nucleoside transporters (Baldwin et al., 2004). Therefore, the generation of intracellular adenosine and its exportation to the external medium occurs when AMP accumulates, giving the nucleoside the possibility to act as a danger signal, both by interacting with specific receptors on the same cell or on the neighboring cells. Intracellular adenosine can be converted into both AMP, by adenosine kinase (ADK) and inosine, by ADA. In turn, AMP, through the sequential action of AMPD and cytosolic NT5C2 is converted into inosine, which, by the action of purine nucleoside phosphorylase (PNP) is transformed into hypoxanthine (Figure 1) (Camici et al., 2018b). Inosine and hypoxanthine can also be transported to the extracellular compartment (Yao et al., 2002). As shown in Figures 1, 3, adenosine can be also generated intracellularly from S-adenosyl homocysteine (SAH) through the action of S-adenosyl homocysteine hydrolase (SAHH) (Garcia-Gil et al., 2018).

ATP can exit the cells through vesicles or through pannexin channels, or can be released by dying cells (Bodin and Burnstock, 2001). The nucleotide can interact with two families of receptors: P2X receptors, which are ion-gated channels displaying neuromodulatory functions, and P2Y receptors, which are G-protein coupled (Burnstock, 2017). This interaction modulates neuronal firing and mediates neuroinflammation (Rodrigues et al., 2015). Extracellularly, ATP can be dephosphorylated to AMP by ectonucleoside triphosphate diphosphohydrolase (CD39). Then, AMP can be dephosphorylated to adenosine by the extracellular 5’-nucleotidase (CD73). Extracellular adenosine can be converted into hypoxanthine and ribose-1 phosphate by the combined action of ectosolic ADA and PNP (Figure 1). In fact, there are several indications that both ADA and PNP are present inside the cells and are also released in the extracellular space ensuring a rapid degradation of extracellular adenosine into hypoxanthine, thereby helping to prevent a dangerous accumulation of the nucleoside. This occurrence has also been reported in the brain (Wall et al., 2007; Gracia et al., 2008). Extracellular ribose-1-phosphate might be dephosphorylated by several phosphatases present on the membrane and equilibrates with the intracellular ribose possibly through one member of the family of glucose transporters as demonstrated in *Leishmania* (Naula et al., 2010).

As a result of the regulation of its metabolism, adenosine does not reach very high concentrations in healthy cells because it is readily metabolized. Hypoxanthine can be salvaged by hypoxanthine-guanine phosphoribosyltransferase (HPRT) into inosine monophosphate (IMP) or can be excreted as uric acid. Ribose- 5- phosphate can be utilized for 5-phosphoribosyl-1-pyrophosphate (PRPP) synthesis, and can be used for energy repletion or glucose synthesis (Garcia-Gil et al., 2018). Indeed, adenosine accumulation can impair essential cellular functions. In fact, deficiency of ADA which brings about an accumulation of adenosine and deoxyadenosine is the most common cause of severe combined immunodeficiency (SCID). These purine compounds are detrimental particularly for the immune system and also impair the functionality of the nervous system (Sauer et al., 2017). In this review, we describe the contribution of 5’-nucleotidases, ADK, ADA, AMPD and nucleoside transporters in epilepsy, cognition, and neurodegenerative diseases with a particular attention on serious pathological conditions such as ALS and HD. For a better insight on adenosine receptor expression, function and regulation, the reader is referred to the numerous excellent reviews covering the different aspects of purinergic receptors (Cunha, 2016; Burnstock, 2017).

## 5’–Nucleotidases

NT5C1, which has been mainly studied in skeletal muscle, and NT5C2, which is ubiquitously expressed, are the major cytosolic NT5Cs acting on intracellular nucleotides (Camici et al., 2020). Among purine nucleotides, AMP, with a K_M_ in the millimolar range (Hunsucker et al., 2001; Tkacz-Stachowska et al., 2005) is the preferred substrate for NT5C1, while IMP and GMP are better substrates for NT5C2 (K_M_ in the micromolar range) (Tozzi et al., 2013; Pesi et al., 2021), but this enzyme catalyzes also the hydrolysis of the phosphoester bond of AMP (with a K_M_ in the millimolar range) (Tozzi et al., 2013). The intracellular concentrations of IMP, GMP and also AMP depend on the rate of the AMP-IMP-GMP cycles (Figure 2) which in turn depends on NT5C2 activity (Barsotti et al., 2003). In fact, at high energy charge, excess IMP, synthesized by *de novo* or salvage pathways, is converted to inosine and therefore directed to catabolic pathways, while at low energy charge IMP and AMP accumulate (Pesi et al., 1994; Allegrini et al., 2004; Wallden and Nordlund, 2011; Camici et al., 2018a). NT5C1 has been associated to some autoimmune diseases (Rietveld et al., 2018), while an association has been reported between NT5C2 expression and psychiatric and psychomotor disorders including schizophrenia (Cross-Disorder Group of the Psychiatric Genomics Consortium, 2013; Duarte et al., 2016; Duarte et al., 2019) and hereditary spastic paraplegias (HSP) (Garcia-Gil et al., 2018; Camici et al., 2020). At early stages of AD, NT5C activity is reduced in membranes and cytosol in distinct cortical regions such as the frontal cortex, and only at advanced stages in cytosol in the temporal cortex (Alonso-Andres et al., 2018). Recently, decrease in NT5C2 activity has also been found in the senescence-accelerated mouse-prone 8, a model of AD (Sanchez-Melgar et al., 2020).

HSPs comprise a group of genetically heterogeneous neurodegenerative disorders presenting progressive spasticity in the lower limbs (Blackstone, 2018). In addition, skin abnormalities, epilepsy, intellectual disability, deafness, optic atrophy, peripheral neuropathy and ataxia, have been reported in association with autosomal recessive inheritance. Several mutations associated with NT5C2 in HSP type 45 have been described (Novarino et al., 2014; Darvish et al., 2017; Elsaid et al., 2017; Straussberg et al., 2017; Naseer et al., 2020). A splice mutation associated with a substantial reduction in NT5C2 level has been found in two children with severe early spasticity, mild cognitive impairment, and dysgenic and thin corpus callosum (Elsaid et al., 2017), whereas corpus callosum with normal white matter was found in their apparently normal heterozygous brother. Therefore, homozygous alteration in NT5C2 appears to be necessary to produce central white matter developmental defects (Elsaid et al., 2017). A 1954-bp homozygous deletion at the *NT5C2* locus involving the entire coding exon 11 was identified in two siblings with HSP and intellectual disability (Darvish et al., 2017). Microcephaly has been found in two cases described by Novarino et al. (2014) and Naseer et al. (2020). The mechanisms underlying the clinical manifestations of NT5C2 mutations are unknown but it has been reported that NT5C2 expression is higher during fetal development and that, within the adult brain, NT5C2 is enriched in neurons compared to glial cells (Duarte et al., 2019). The same authors have demonstrated that *NT5C2* knockdown in human neural stem cells increased the expression and the phosphorylation of the α−subunit of AMPK (Duarte et al., 2019). Moreover, studies in *Drosophila melanogaster* have shown that knockdown of the *NT5C2* homologue in neurons is associated with climbing impairment suggesting a role for NT5C2 in motility (Duarte et al., 2019). Furthermore, we demonstrated that transitory *NT5C2* silencing in an astrocytoma cell line (ADF) caused apoptosis, while a constitutive silencing increased oxidative metabolism and decreased cell proliferation (Careddu et al., 2008; Pesi et al., 2018). It would be valuable to obtain information on the levels of expression and activity of NT5C2 and the concentration of AMP and ATP in HSP patients, since alterations of their ratio affect AMPK activity, and a continous activation of this enzyme could result in abnormal functioning and development of the nervous system (Pesi et al., 2000; Garcia-Gil et al., 2003; Williams et al., 2011; Domise et al., 2019) (see section AMPK).

In humans, the family of NT5C2 is encoded by five genes (*NT5C2, NT5DC1-4*). The presence of a nucleotidase domain in *NT5DC1-4*, allows to hypothesize that these genes code for proteins having 5’-nucleotidase activity, but the enzymatic activity has not been directly measured. Singgih et al. (2021) have recently reported the expression of orthologues of the NT5C2 family in the brain of *Drosophila melanogaster* and that the neuronal knockdown of two of them resulted in impaired habituation learning. *NT5DC2* has also been associated with cognitive ability, bipolar disorder and schizophrenia (Watanabe et al., 2019) while the expression level of *Nt5dc3*, has been positively correlated with reversal learning performance in mice (Laughlin et al., 2011).

## Ectonucleotidases

Ectonucleotidases control the levels of ATP and its hydrolysis products ADP, AMP and adenosine in the synaptic cleft. The major families of ectonucleotidases are CD39, ecto-nucleotide pyrophosphatase/phosphodiesterases, alkaline phosphatases, and CD73. Adenosine, the final product of ATP extracellular degradation, can be either degraded by extracellular ADA and PNP into hypoxanthine and ribose-1-phosphate, or enter the cell through equilibrative and/or concentrative nucleoside transporters (see below, nucleoside transporters section) (Figure 1). CD73 is inhibited by micromolar concentration of ADP and ATP (Zimmermann, 1992), therefore when ATP is released and accumulates outside the cell, it is hydrolyzed into ADP and AMP and is converted to adenosine only after the disappearance of ATP and ADP. In the basal ganglia, CD73, which generates adenosine from extracellular AMP, colocalizes with A2ARs (Augusto et al., 2013) and the enzyme has been recently demonstrated to act a part in A2AR signaling in both PD models and patients (Carmo et al., 2019; Meng et al., 2019). CD73-derived adenosine-A2AR signaling is able to modulate microglial immunoresponses and the extension of microglial processes and movement (Meng et al., 2019). Moreover, the reduction of adenosine generated from CD73 decreased microglia-mediated neuroinflammation, increased dopaminergic neuron viability and motor function in a model of PD (Meng et al., 2019). In a model of AD, β-amyloid increased ATP release and CD73 activity, leading to adenosine generation, activation of A2AR and impairment of synaptic plasticity and memory (Goncalves et al., 2019). The hippocampal astrogliosis observed in mesial temporal lobe epilepsy patients is associated to an increase in expression of A2AR and CD73 (Barros-Barbosa et al., 2016). Finally, in addition to its role in the generation of adenosine, CD73 might participate in the regulation of cell adhesion, migration and differentiation, since CD73 acts as receptor for extracellular matrix molecules including tenascin C, fibronectin and laminin (Sadej et al., 2008).

In many cases neurodegenerative diseases are the consequence of neuroinflammation. In reactive astrocytes, increased *CD73* gene expression or increased CD73 activity was reported in ALS (Gandelman et al., 2010), epilepsy (Bonan et al., 2000; Bonan, 2012), ischemia (Braun et al., 1998) and traumatic brain injury (Nedeljkovic et al., 2006; Bjelobaba et al., 2011). Upregulation of CD73 has also been found in glioma (Quezada et al., 2013; Xu et al., 2013), while in a model of multiple sclerosis, upregulation of CD73 and CD39 has been reported in reactive astrocytes and in microglia, respectively (Lavrnja et al., 2015; Jakovljevic et al., 2019). The involvement of ectonucleotidases in the regulation of microglial function, the pathogenesis of infectious diseases in the nervous system and the complex regulation of CD73 at the neurovascular unit during neuroinflammation have been recently reviewed by Nedeljkovic (2019), Alves et al. (2020) and Illes et al. (2020). Altogether these observations suggest that reduction of CD73 activity, and therefore, adenosine levels, might be a therapeutical tool to decrease neuroinflammation in PD as well as astrogliosis in mesial temporal lobe epilepsy, and likely, to improve cognition in AD and PD.

## Adenosine Kinase

ADK catalyzes the transfer of the γ-phosphate from ATP to adenosine, leading to the formation of AMP, and has the capability to regulate both extracellular adenosine and intracellular adenine nucleotide levels. Human ADK consists of two alternatively spliced forms with distinct cellular and subcellular localization. ADK-short (ADK_S_) is mainly cytoplasmatic and the longer form (ADK_L_) is nuclear (Cui et al., 2009; Kiese et al., 2016; Boison and Jarvis, 2020). ADK is found mainly in neurons at early stages of development but later it becomes more abundant in astrocytes. The function of the two isoforms appears to be different. Indeed, overexpression of ADK_S_ in the brain resulted in spontaneous seizures and increased brain injury after ischemic stroke (Li et al., 2008; Shen et al., 2011). Overexpression of ADK_L_ in dorsal forebrain neurons attenuated neural stem cell proliferation (Gebril et al., 2020), while transgenic mice lacking ADK_L_ in the dental gyrus showed increased neurogenesis. In addition, ADK_L_ might have a more prominent role in epigenetic mechanisms requiring transmethylation than ADK_S_ (Williams-Karnesky et al., 2013). Methyltransferases catalyze the transfer of methyl groups from the donor S-adenosylmethionine (SAM) to proteins such as histones and DNA yielding S-adenosylhomocysteine (SAH) (Figure 3), whose cleavage into adenosine and homocysteine is catalyzed by S-adenosylhomocysteine hydrolase (SAHH). The removal of adenosine by ADK favors the transmethylation reactions. When the clearance of adenosine is impaired, the increased levels of SAH inhibit DNA methyltransferases (James et al., 2002) (Figure 3).

Both deficiency and excess of ADK are harmful. ADK deficiency is a very rare inborn error of metabolism, and is characterized by defects in transmethylation reactions associated with developmental delay, hepatic encephalopaties as well as seizures in some individuals (Bjursell et al., 2011; Shakiba et al., 2016; Staufner et al., 2016; Alhusani et al., 2019; Becker et al., 2021). Mutations of ADK or modification of ADK levels have been associated with several diseases (Garcia-Gil et al., 2018), such as stroke (Shen et al., 2011), Rasmussen encephalitis (Luan et al., 2013), focal cortical dysplasia (Luan et al., 2015), epilepsy (Boison, 2016) and gliomas (de Groot et al., 2012; Huang et al., 2015), as well as in cognition deficits (Bjursell et al., 2011; Singer et al., 2012; Sandau et al., 2016; Shakiba et al., 2016; Staufner et al., 2016; Osborne et al., 2018; Kuptanon et al., 2019). Notably, genetic variants of ADK are associated with post-traumatic epilepsy in humans (Diamond et al., 2015). Dysfunction of adenosine signaling, which is common in neurological disorders, might explain comorbid phenotypes such as epilepsy, PD, ALS and AD among others (Boison and Aronica, 2015).

Inhibition of ADK strengthens A1AR activation and has a protective effect in ischemia, epilepsy and glutamate excitotoxicity (Boison, 2013). Williams-Karnesky et al. (2013) have demonstrated that epileptogenesis is modulated by intracellular adenosine through the transmethylation pathway. Indeed, seizures in murine models involve alteration of adenosine homeostasis (increased ADK and reduced adenosine), increased DNA methyltransferase activity and increased hippocampal DNA methylation (Williams-Karnesky et al., 2013). Seizure susceptibility was reduced by DNA methyltransferase inhibitors (Williams-Karnesky et al., 2013). Different methods are being tried to increase adenosine in order to reduce seizures. When adenosine-releasing polymers were implanted intraventricularly, methylation reverted to control levels and seizure activity decreased (Williams-Karnesky et al., 2013). Recently, attenuation of epilepsy development in mice has also been obtained after transient application of an ADK inhibitor (Sandau et al., 2019). ADK-deficient stem cells could be a tool to increase adenosine. Poppe et al. (2018) have obtained ADK-deficient epithelial stem cells able to differentiate in neurons and astrocytes with high ability to release adenosine. ADK levels are reduced by ketogenic diet used for the treatment of epilepsy. It is interesting to note that the ketogenic diet suppresses seizures by A1R-dependent and likely also by adenosine-dependent epigenetic mechanisms (Masino et al., 2011; Lusardi et al., 2015; Boison and Rho, 2020).

Transgenic mice with brain-wide or telencephalon ADK hyperexpression showed dysregulation of brain adenosine which resulted in impairment of working memory and of associative memory measured using the conditioned freezing paradigm (Yee et al., 2007; Singer et al., 2012; Singer et al., 2013), while mice with brain-wide deletion of ADK developed spontaneous seizures and profound deficits in hippocampus-dependent learning and memory (Sandau et al., 2016).

Recently, Osborne et al. (2018) have distinguished the neurobehavioral consequences of gestational ADK deletion vs. adult-onset ADK deficiency. Interestingly, gestational depletion of ADK produced deficits in social memory in males, and contextual learning impairments in both sexes, and a hyper-responsiveness to amphetamine in males. In contrast, the tardive astrocyte deficiency of ADK resulted in normal social memory and contextual learning. These results point to a role for adenosine homeostasis during development in the determination of the susceptibility to later neuropsychiatric diseases such as schizophrenia, autism and attention deficit hyperactivity disorder, in which males more frequently express social deficits than females.

Clinical radiation therapy for the treatment of central nervous system tumors leads to impairments in cognition. Adult rats exposed to cranial irradiation showed significant declines in performance of hippocampal-dependent memory tasks such as novel place recognition, novel object recognition and contextual fear conditioning associated to astrogliosis and elevated ADK expression in the hippocampus. The treatment with an ADK inhibitor prior to cranial irradiation improved performance in all cognitive tasks one month post exposure (Acharya et al., 2016).

## Adenosine Deaminase

ADA catalyzes the deamination of adenosine and deoxyadenosine into inosine a deoxyinosine respectively. There are two genes codifying for ADA: *ADA1* and *ADA2*/cat eye syndrome chromosome region, candidate 1 (*CECR1*) which are localized in the chromosomes 20 and 22, respectively. The proteins have 27% identity and differ in structure and probably in functions (for recent reviews see Meyts and Aksentijevich, 2018; Moens et al., 2019).

### Adenosine deaminase 1

ADA1 is a 41-kDa monomer protein that is present in all human tissues, with the highest expression in T and B lymphocytes. ADA1 plays a crucial role in adaptive immune system development and exhibits an affinity for its substrates adenosine and deoxyadenosine significantly higher than ADA2. ADA1 not only reduces extracellular adenosine concentration preventing adenosine receptor desensitization, but is also able to directly interact with dipeptidyl peptidase-4 (CD26) (Franco et al., 1997) and, by interacting with A1R and A2R, increases receptor functionality in the striatum (Gracia et al., 2008; Ciruela et al., 2010; Gracia et al., 2011). CD26 is more expressed in microglia and astrocytes than in neurons and is more abundant in astrocytes during inflammation and in microglia in neuropathy. Spinal application of CD26 inhibitors induces a strong antihyperalgesic effect during inflammatory pain (Kiraly et al., 2018), but the role of ADA1 in CD26 function in the nervous system is unknown.

Moreno et al. (2018) have demonstrated the formation of trimeric complexes CD26-ADA-A2AR and have suggested that ADA could have a role in communication between cells expressing CD26 (such as T cells) and those expressing adenosine receptors (such as neurons and dendritic cells). If this is the case, ADA deficiency could also affect the cell-cell communication in the nervous system.

Mutations in the *ADA1* gene are among the most common causes for severe combined immunodeficiency (SCID). When ADA activity is absent, deoxyadenosine increases both extracellularly and intracellularly. Within cells, it is converted by the action of deoxycytidine kinase and/or adenosine kinase to deoxyadenosine monophosphate and then to deoxyadenosine triphosphate (dATP). Deoxyadenosine and dATP in lymphocytes are considered the main agents of toxicity. The raise of intracellular dATP interferes with DNA synthesis and repair by inhibiting ribonucleotide reductase and terminal deoxynucleotidyl transferase and results in apoptosis of developing thymocytes (for recent reviews Whitmore and Gaspar, 2016; Flinn and Gennery, 2018; Garcia-Gil et al., 2018; Camici et al., 2019) while deoxyadenosine inactivates SAHH, leading to accumulation of SAH and inhibits the transmethylation reactions (Figure 3) which are required for lymphocyte activation.

In addition to immunodeficiency, ADA-SCID patients display skeletal, hepatic, renal and lung alterations, as well as neurological and behavioral impairments such as reduced verbal expression, seizures, learning disability, hyperactivity, attention and hearing deficits (Rogers et al., 2001; Nofech-Mozes et al., 2007; Titman et al., 2008; Garcia-Gil et al., 2018). The accumulation of adenosine and deoxyadenosine caused by ADA deficiency might contribute to the alterations in the nervous system. Notably, a polymorphism in the *ADA1* gene in autistic children with mild intellectual disability has been found to be associated with reduced ADA activity in serum (Stubbs et al., 1982; Bottini et al., 2001; Saccucci et al., 2006). Neurological deficits persist after bone marrow transplant or replacement therapy which do improve the immunological and metabolic aspects of the disease (Honig et al., 2007; Booth and Gaspar, 2009; Cicalese et al., 2016). Recently, the degree of neurological impairment of *Ada−/−* mouse treated with PEG-ADA has been compared with untreated controls (Sauer et al., 2017). The knockout mice show undetectable ADA activity, increased adenosine in total brain extracts, slightly reduced brain size, alterations in explorative behavior, increased anxiety, reduced pain sensitivity and normal sensorimotor development. Both untreated and PEG-ADA-treated knockout mice exhibited reduced A2AR level compared to control brains, suggesting that adenosine signaling is affected by ADA deficiency. Adenosine metabolite levels in the brain, ventriculomegaly and pain sensitivity showed a tendency to decrease after PEG-ADA treatment (Sauer et al., 2017) while exploration and anxiety abnormalities remained uncorrected. Therefore, reduction of A2AR appears to contribute to the phenotype of the *Ada−/−* mice but the involvement of other mechanisms, such as epigenetic alterations have not been addressed. Interestingly, it has been recently reported that when the enzyme replacement therapy with PEG-ADA is performed early, it is effective in improving hearing defects in *Ada−/−* mice (Xu et al., 2019). ADA enzyme therapy in these mice normalized cochlear adenosine levels, and prevented demyelination while treatment with an A2BR-antagonist improved hearing loss and myelin compaction (Manalo et al., 2020).

### Adenosine deaminase 2

ADA2 has a 100-fold higher K_M_ for adenosine (2 mM) than ADA1 (Schrader et al., 1978). Therefore, the deaminase activity of ADA2 is low under physiological conditions but could be relevant during inflammation and tumorigenesis, when adenosine levels increase. For recent reviews see Meyts and Aksentijevich (2018) and Moens et al. (2019).

Deficiency of ADA2 arises from mutations affecting catalytic activity, protein dimerization, and secretion of ADA2 and causes vasculopathy and inflammation in many organs and/or hemorrhagic stroke (Navon Elkan et al., 2014; Zhou et al., 2014; Meyts and Aksentijevich, 2018; Gibson et al., 2019; Sahin et al., 2020; Zervou et al., 2020; Sozeri et al., 2021). Less frequent neurological manifestations include spastic diplegia or paraplegia, peripheral polyneuropathy, ataxia, neurosensory deafness, and cerebral atrophy (Meyts and Aksentijevich, 2018). ADA1 activity is not impaired and deoxyadenosine nucleotides do not accumulate in ADA2 deficiency (Meyts and Aksentijevich, 2018).

In addition to its deaminase activity, ADA2 may have a growth factor activity and contrarily to ADA1, does not bind to CD26. ADA2 is a potent regulator of tumor associated microglia/macrophages polarization. It has been found highly expressed by tumor associated microglia/macrophages (M2-type) in high-grade glioma. In these cells, paracrine effects induced by ADA2 include activation of MAPK signaling and stimulation of proliferation and migration of glioma cells (Zhu et al., 2017a). Moreover, ADA2/CECR1 mediates cross-talk between macrophages and pericytes in glioblastoma multiforme resulting in pericyte recruitment and migration, and thus promoting tumor angiogenesis (Zhu et al., 2017b).

## Amp Deaminase

AMPD converts AMP into IMP by deamination (Figures 1, 2). Together with CD39 and NT5C, it regulates the purine pool of nucleotides. The three genes coding for AMPD, AMPD1, AMPD2, and AMPD3, are differently expressed in various organs and in various types of cells. AMPD1 is highly expressed in skeletal muscle and diaphragm, AMPD2 is mainly expressed in brain, liver, and thymus and AMPD3 is most strongly expressed in erythrocytes (Morisaki et al., 1990; Mahnke-Zizelman and Sabina, 1992). Mutations in AMPD2 result in pontocerebellar hypoplasia due to loss of brainstem and cerebellar parenchyma (Akizu et al., 2013; Marsh et al., 2015; Accogli et al., 2017; Kortum et al., 2018; Abreu et al., 2020) but a homozygous *AMPD2* frameshift variant has been associated with HSP type 63 (Novarino et al., 2014). AMPD2 plays a role in guanine nucleotide homeostasis by regulating the feedback inhibition of adenosine derivatives on *de novo* purine synthesis; AMPD2 deficiency results in increase of ATP and decrease of GTP levels, which leads to impairment of GTP-dependent initiation of protein synthesis (Akizu et al., 2013). Recently, *AMPD1* polymorphisms have been associated to autism risk in Chinese population (Zhang et al., 2015). Studies performed in lymphoblast cell lines obtained from patients have revealed decreased mitochondrial complex I activity compared to control cells. This result is interesting since reduction of transcription of mitochondrial electron transport complex genes has been found in several regions of autism brains (Anitha et al., 2013). Since AMPD1 is particularly enriched in muscle, it would be interesting to study the effects of these variants on the muscle functionality of the autistic patients.

## Amp-Activated Protein Kinase

AMPK is the principal regulator of cellular energy homeostasis since it is a sensor of the AMP:ATP ratio and mediates the adaptive changes of the cell as a function of the energy charge. In fact, through complex and various signaling pathways, AMPK switches off the anabolic pathways that require ATP and switches on the catabolic pathways that produce ATP (Hardie and Hawley, 2001; Gowans et al., 2013). Increasing evidence supports a relevant role of AMPK in the physiopathology of the central nervous system (Camici et al., 2020). The roles of AMPK in brain and its cross-talk with many hormones in the hypothalamus to mediate their anorexigenic and orexigenic effects as well as thermogenic influences have been thoroughly reviewed (Huynh et al., 2016; Rosso et al., 2016; Peixoto et al., 2017; Liu et al., 2020).

Constant AMPK activation might lead to abnormal functioning and development of the nervous system. We have reported that AMPK activation promotes apoptosis in hippocampal and neuroblastoma cells (Pesi et al., 2000; Garcia-Gil et al., 2003) while other authors have described reduction of axonal growth (Williams et al., 2011) and reduction of synapses (Domise et al., 2019). In addition, it has been reported that dysregulation of the AMPK signaling in motor neurons is an early and common event in ALS (Perera and Turner, 2016) and that psychiatric disorders are also associated with dysregulation of AMPK signaling (Perera and Turner, 2016; Rosso et al., 2016). In particular, it has recently been found that AMPKα1 levels are significantly increased, while AMPKα2 levels are markedly reduced in the hippocampus of AD patients, compared to controls (Zimmermann et al., 2020). These changes in AMPKα expression appeared to be AD specific, since AMPKα1/2 levels were unaffected in either Lewy body dementia or frontotemporal dementia (Zimmermann et al., 2020).

Many research groups have studied the effect of AMPK activation on the development of AD, but the results are controversial. When the effect of metformin, a known activator of AMPK and a drug used for the treatment of type 2 diabetes, has been investigated on cognition deficits, some studies have shown an aggravating effect (Imfeld et al., 2012; Wennberg et al., 2018), whereas others reported a preventive one (Ng et al., 2014; Shi et al., 2019). Metformin has been shown to have a positive effect on AD mouse model (Wang et al., 2020). Recently it has been investigated the possible role of the AMPK isozymes in these conflicting results using mouse models of AD (Zimmermann et al., 2020). *AMPKα2* but not *AMPKα1* knockout mice displayed impaired cognition and hippocampal late long-term potentiation (Yang et al., 2020). In contrast, the brain-specific repression of AMPKα1 attenuated learning and memory deficits and the synaptic failure in mouse models of AD (Zimmermann et al., 2020). Moreover, AD-associated abnormal eEF2 phosphorylation and *de novo* protein synthesis reduction were also alleviated by deletion of the AMPKα1 isoform. This effect is relevant for cognition, since long-term memory requires new protein synthesis (Alberini, 2008). The effects of AMPK on amyloid β peptide accumulation, tau aggregation, and oxidative stress have been recently reviewed (Assefa et al., 2020). The role of AMPK in HD and ALS is discussed later in the appropriate sections.

## Nucleoside Transporters in the Brain

As described in the previous sections, adenosine is produced both extra- and intracellularly and is transported across the cell membrane. Therefore, nucleoside transporters have a major impact on the adenosine level, both inside and outside the cell. The *SLC28* gene family encodes the concentrative Na^+^-dependent nucleoside transporters (CNT1-3), while the *SLC29* gene family encodes the Na^+^-independent equilibrative nucleoside transporters (ENT1-4) (Cabrita et al., 2002; Engel et al., 2004; Zhou et al., 2010). Among the concentrative transporters, CNT1, which prefers transport of purine nucleosides, and CNT2, which shows a broader substrate specificity, accepting both purine and pyrimidine nucleosides, are expressed in the brain (Anderson et al., 1996). Although all ENTs have been found in the brain (Anderson et al., 1999a; Anderson et al., 1999b; Engel et al., 2004; Baldwin et al., 2005) only ENT1 and ENT2 appear to be relevant in the purinergic signaling.

### Concentrative Transporters

CNTs are high-affinity inward transporters for adenosine. CNT1 is the first nucleoside transporter defined as a transceptor (Pérez-Torras et al., 2013), a word deriving from the contraction of **trans**porter and re**ceptor** for the coexistence of these two functions (Pastor-Anglada and Pérez-Torras, 2018). The expression of the highly regulated transporter CNT1 (Valdes et al., 2002; Klein et al., 2009), is reduced in several human tumors (Farre et al., 2004; Bhutia et al., 2011). Pérez-Torras et al. (2013) found that the restoration of human CNT1 in pancreatic cancer cells caused an alteration in cell cycle progression and in the phosphorylation status of kinases involved in key signaling pathways, promoted polyADPR polymerase activity and non-apoptotic cell death, and decreased cell migration, thus reducing tumor growth. All these effects were mimicked by a translocation-defective human CNT1 variant, indicating that transport is not required for signaling. Although the regulation of CNT1 appears extremely interesting and relevant in tumor biology, its involvement in the effect of adenosine in brain still remains unclear.

CNT2 has been reported to be under the A1R control in hepatocytes (Duflot et al., 2004). A similar cross-talk between CNT2 and A1R appears to operate also in neurons since *in situ* hybridization in rat brain demonstrated that the prominent expression areas of CNT2 are rich in A1R (Guillén-Gomez et al., 2004; Pastor-Anglada and Pérez-Torras, 2018). Nerve growth factor-induced differentiation in phaeochromocytoma PC12 cells increased progressively CNT2 expression and raised A1R mRNA by 15-fold (Medina-Pulido et al., 2013). Although triggering antagonistic signals in target cells, A1R and A2AR significantly up-regulated CNT2 transport activity, both promoting removal of extracellular adenosine in differentiated neuronal PC12 cells (Medina-Pulido et al., 2013). In an intestinal rat epithelial cell line, through a CNT2 and ADK-mediated mechanism, the addition of adenosine rapidly increased AMP intracellular concentration with a consequent activation of AMPK (Aymerich et al., 2006). The same adenosine-dependent AMPK activation has been reported in differentiated neuronal PC12 cells. Interestingly, hypoxia induced a down-regulation of CNT2 and a consequent inactivation of AMPK (Medina-Pulido et al., 2013). Therefore, a functional link between CNT2-mediated adenosine uptake and energy metabolism appears to be present not only in intestinal cells (Aymerich et al., 2006; Huber-Ruano et al., 2010), but also in neuronal cells (Medina-Pulido et al., 2013; Pastor-Anglada and Pérez-Torras, 2018). Brain extracellular adenosine in cats increases during wakefulness and decreases during the spontaneous recovery sleep. The duration and depth of sleep after wakefulness appear to be modulated by adenosine (Porkka-Heiskanen et al., 1997). Guillén-Gomez et al. (2004) demonstrated that total sleep deprivation, which is accompanied by an increase of extracellular adenosine, decreased the amount of CNT2 mRNA in the rat cerebral cortex. Therefore, CNT2 expression appears to be regulated by sleep at transcriptional level. Since CNT2 is far more efficient in the uptake of adenosine than any equilibrative transporter (Parkinson et al., 2011), its specific decrease suggests that this transporter may exert a new physiological role for this transporter in the modulation of extracellular adenosine levels and the sleep/wakefulness cycle.

### Equilibrative Transporters

ENTs, contrary to CNTs, are widely distributed in all tissues, including the central nervous system, and are considered important components of the purinergic signaling in brain (Pastor-Anglada and Pérez-Torras, 2018). Most studies focus on ENT1 and ENT2; indeed, ENT3 is distributed in intracellular membranes (Baldwin et al., 2005), while ENT4 cannot be considered a conventional nucleoside transporter, but rather a polyspecific cation transporter (Engel et al., 2004). Alterations in the function of ENTs bring about modified levels of adenosine, thus resulting in aberrant purinergic signaling. Mice lacking ENT1 exhibit dysfunctional behaviors, such as decreased ethanol intoxication and excessive ethanol drinking (Choi et al., 2004; Chen et al., 2010), and reduced anxiety-like behavior (Chen et al., 2007; Ruby et al., 2011; Nam et al., 2013). In the dorsomedial striatum of ENT1 null mice, a correlation between higher ethanol consumption and a decreased adenosine-mediated A2AR signaling has been demonstrated (Nam et al., 2013). On the other hand, the lack of ENT1 or the inhibition of ENT1 in the amygdala, leads to an increased adenosine-mediated A1R signaling, which correlates with a reduced anxiety behavior (Choi et al., 2004; Chen et al., 2007). Indeed, since ENT1 mediates nucleoside transport bidirectionally depending on the concentration gradient across the membrane, Ruby et al. (2011) hypothesize that ENT1 mediates a release of adenosine in the striatum (Ruby et al., 2011; Nam et al., 2013), while in the amygdala, ENT1 primarily mediates uptake of adenosine (Choi et al., 2004; Chen et al., 2010; Ruby et al., 2011). In this regard, an important bias of all these studies is the lack of a reliable technique allowing for the detection of adenosine. In fact, the changes in adenosine concentrations have been inferred, but never directly measured.

A correlation between ENT1 expression and epilepsy severity has been reported in rats (Xu et al., 2015; Zhou et al., 2020). In several epilepsy models, adenosine has been shown to exert an anticonvulsant effect (Boison, 2012; Masino et al., 2014), which appears to be mainly mediated by A1R (Li et al., 2007). ENT1 may be considered a therapeutic target for the control of epileptic seizures (Huang et al., 2017). Indeed, intraperitoneal and hippocampal injections of nitrobenzythioinosine (NBTI), a specific inhibitor of ENT1, decreased the number of seizures in rats (Xu et al., 2015), while blood brain barrier permeable ENT1 inhibitors have shown anti-epileptic effects in different mouse models (Ho et al., 2020). Recently, Zhou et al. (2020) demonstrated that, besides NBTI, also the specific inhibition of the p38 MAPK signaling pathway in the brain tissues of rats with acute status epilepticus, reduced the expression levels of ENT1 as well as A1R, the expression of which was significantly increased after seizure induction, probably as adaptive response to an acute attack. The p38 MAPK signaling inhibition decreased pathological damage of hippocampal neurons and reduced frequency of seizures. However, further investigation is needed to clarify the molecular mechanisms underlying the observed effects (Zhou et al., 2020). The expression of ENT1 in HD models and patients is discussed below.

Increasing adenosine by using an ENT1 inhibitor, improved memory deficts in a mouse model of AD (Lee et al., 2018). Studies in non-mammal models have also suggested a role for adenosine signaling and metabolism in learning. For example, antagonists of adenosine receptors, and inhibitors of ENT and ADA were able to prevent the scopolamine-induced amnesia in zebrafish (Bortolotto et al., 2015).

Adenosine has been reported to aberrantly increase in glioblastoma tumors, and the adenosine signaling has been associated with increased chemoresistance, migration and invasion, especially in a cell population with an extremely aggressive behavior, called glioblastoma stem-like cells (GSCs) (Niechi et al., 2019; Torres et al., 2019). Recently, Alarcon et al. (2020), using a HPLC technique with fluorescent detection (Torres et al., 2016), demonstrated that extracellular adenosine was significantly increased in GSCs as compared to non-GSCs and that this difference was specifically ascribable to the mesenchymal GSC subtype. A significant reduction in the uptake of adenosine was observed in GSCs as compared to non-GSCs, although mRNA and protein levels of ENT1 were not significantly different (Alarcon et al., 2020). Therefore, post-translational modifications of the transporter may be relevant in the alteration of its transport efficiency. In this regard, ENT1 has been shown to be regulated by kinase-dependent pathways (Bone et al., 2007; Reyes et al., 2011), and, as discussed below, also protein-protein interaction might contribute to the activity of ENT1.

Although mRNA for ENT2 has been reported to be expressed in brain (Anderson et al., 1999a), ENT2 was suggested to play a major role in the regulation of adenosine levels in the gastrointestinal tract (Morote-Garcia et al., 2009; Pastor-Anglada and Pérez-Torras, 2018). Its function for the uptake of purine analogs for chemotherapic treatment of various cancer types and its involvement in the cell cycle progression have been recently reviewed by Naes et al. (2020).

### Nucleoside Transporter Interactome

Nucleoside transporters have been considered as independent entities that regulate the traffic of nucleosides across the membrane. However, growing lines of evidence indicate that they may interact with other proteins, therefore belonging to a more complex network (Dos Santos-Rodrigues et al., 2014). For example, glucose-regulated protein 58 (GRP58) and aldolase B were identified as possible partners of CNT2 (Huber-Ruano et al., 2010), therefore a regulatory model dependent on nutrient availability can be postulated for CNT2. Although not yet described, the transceptor role of CNT1 might imply the occurrence of interactions with other proteins, which might explain its function apart from that of nucleoside transporter. Concerning ENT1, the only interactor confirmed so far is calmodulin (Bicket et al., 2016), which could explain, at least in part, the calcium-dependent release of nucleosides in neural cells (Zamzow et al., 2009).

## Huntington’s Disease

HD is a neurodegenerative disorder that is caused by expanded CAG repeats within the exon-1 of the gene coding for huntingtin (HTT). CAG repeat lengths of up to 34 are considered to be physiological, while longer CAG repeats are associated to the development of HD. The age of disease onset correlates inversely with CAG repeat length. HD is characterized by involuntary, abnormal movements and postures, psychiatric disturbances, and cognitive alterations (Bates et al., 2015). Research has been performed in cellular and animal models expressing different lengths of CAG repeats (Table 1). For example, the R6/2 mouse expresses exon 1 of the human *HTT*; the zQ175 mouse expresses a *HTT* carrying 188 CAG repeats, the Tg51 rat expresses a fragment of the *HTT* gene with 51 CAG repeats, while Hdh 150Q is a knock-in mouse expressing the mouse *htt* gene with 150 CAG repeats (Chou et al., 2005; Li et al., 2015; Lee and Chern, 2014; Guitart et al., 2016; Kao et al., 2017). Downregulation of A2AR has been reported in HD rodent models and also in early stages of the disease in humans (Lee and Chern, 2014). Recent studies suggest that reduced extracellular nucleotide breakdown and reduced glycolysis might contribute to the pathology of HD (Toczek et al., 2018; HD iPSC Consortium, 2020) and that ENT1 could be a therapeutical target for this disease (Kao et al., 2017). Measures of intracellular concentration of ATP and NAD^+^, and of the activities of enzymes involved in nucleotide catabolism were performed in the human embryonic kidney cell line HEK 293T transfected with plasmids expressing wild-type or mutant *HTT* gene. Reduction of intracellular ATP, together with increased ADA activity and reduced activities of ectonucleoside triphosphate diphosphohydrolase, ecto-5′-nucleotidase and ectosolic ADA, with no changes of AMPD and PNP activities were observed (Toczek et al., 2018). The authors suggested that mutated HTT could interact with and suppress the activity of the extracellular enzymes involved in nucleotide catabolism thereby contributing to HD pathology (Toczek et al., 2018). More recently, human astrocytes and striatal neurons have been obtained from pluripotent stem cells (iPSCs) derived from unaffected individuals and HD patients with *HTT* gene containing increased number of CAG repeats and used to study the effect of mutant HTT on bioenergetics (HD iPSC Consortium, 2020; Hamilton et al., 2020). While the neurons and astrocytes obtained from iPSCs of control individuals and HD patients had similar levels of ADP and ATP and comparable respiratory and glycolytic activities (Hamilton et al., 2020), the neurons with longer CAG tails, reflecting a more advanced stage of the disease (HD iPSC Consortium, 2020), showed decreased ATP levels and reduced expression of glycolytic enzymes compared to controls. In addition, ATP levels in these HD neurons could be rescued by addition of pyruvate suggesting that the glycolytic deficits play a role in the metabolic disturbance of HD neurons (HD iPSC Consortium, 2020). It would be interesting to test whether the reduced breakdown of ATP takes place in the striatum of HD patients, since this could result in decreased extracellular adenosine and therefore decreased stimulation of the A2AR, which is highly expressed in the striatum and which plays a protective role in this region. Indeed, selective agonists of A2AR reduced DNA damage and oxidative stress-induced apoptosis in HD-iPSC-derived neurons through a cAMP/PKA-dependent pathway (Chiu et al., 2015) and improved motor deficits in mice models of HD (Chou et al., 2005) (Table 1). A role of heteromers of adenosine receptors in HD has been hypothesized but experimental evidence has not yet been provided (Glaser et al., 2020). The decline in energy metabolism observed in HD may be caused both by a gain-of-function of the mutated HTT protein and also by the loss of HTT function. The latter has been demonstrated in cardiomyocytes in which *HTT* was knocked out by the CRISPR/Cas9 method which showed reduced intracellular ATP and reduced cellular medium concentration of total purine pool (Tomczyk et al., 2020).

**TABLE 1:** Involvement of adenosine metabolism enzymes, transporters and receptors in HD.

Enzyme/transporter/receptor	Treatment	Model	Expression/activity	Effect	References
ADA		HEK 293T cells expressing HTT with 54 repeats	Increased activity		Toczek et al. (2018)
Ectonucleoside triphosphate diphosphohydrolase, CD73, ectosolic ADA		HEK 293T cells expressing HTT with 54 repeats	Decreased activity		Toczek et al. (2018)
ADA, CD39, CD73		R 6/2 mouse	No change in expression		Kao et al. (2017)
ADK		R 6/2 mouse	Increased expression		Kao et al. (2017)
ADK		Hdh150Q mouse	No change in expression		Kao et al. (2017)
AMPK		Human,Mouse striatal neurons	Increased expression		Ju et al. (2011)
AMPK	Metformin	R 6/2 mouse		Increased survival	Ma et al. (2007)
AMPK	Metformin	Immortalized striatal cells		Increased survival	Jin et al. (2016)
ENT1		zQ175, R6/2 and Hdh150Q	Increased expression		Guitart et al. (2016)
					Kao et al. (2017)
ENT1	Inhibitor, knockout	R 6/2 mouse		Increased survival, increased motor function	Kao et al. (2017)
ENT2		R6/2 and Hdh150Q mouse	Increased expression		Kao et al. (2017)
A2AR		Human, rat and mouse models	Decreased expression		Lee and Chern (2014)
A2AR		zQ175 mouse	Decreased expression		Guitart et al. (2016)
A2AR		Tg51 rat	No change in expression		Guitart et al. (2016)
A2AR	Agonist	R 6/2 mouse		Decreased motor deficits	Chou et al. (2005)
A2AR	Knockout	R 6/2 mouse		Increased cognitive function	Li et al. (2015)
AR	Caffeine (non selective antagonist)	Human		Decreased disease onset	Simonin et al. (2013)
ARAR gene polymorphisms		Human		Decreased disease onset	Dhaenens et al. (2009); Taherzadeh-Fard et al. (2010)
A1AR		Human	Decreased in symptomatic, increased in presymptomatic		Matusch et al. (2014)
A1AR	Agonist	Rat, 3-nitropropionic acid infusion		Attenuation motor deficit	Blum et al. (2002)
A1AR	Antagonist	R 6/2 mouse	Decreased binding, increased functionality		Ferrante et al. (2014)

Decrease of A2AR has been found in several but not in all HD animal models (Lee and Chern, 2014; Guitart et al., 2016). For example, the number of A2AR antagonist binding sites was not changed in transgenic HD rats with 51 CAG repeats (Tg51 rats). The extracellular level of adenosine measured by *in vivo* microdialysis was lower in rats with 51 CAG repeats and in mice with 175 CAG repeats compared to their wild type littermates (Guitart et al., 2016). Striatal density of ENT1 expression and the expression of ENT1 transcript were significantly increased in mouse expressing HTT with 175 repeats and in postmortem prefrontal cortex from HD patients, respectively (Guitart et al., 2016). Moreover, the ratio of adenosine/ATP in the cerebral spinal fluid was negatively correlated with the disease duration. Chronic inhibition of ENT1 or genetic removal of ENT1 enhanced the survival of mice which express exon 1 of the human *HTT* gene, containing 150 CAG repeats (Kao et al., 2017) (Table 1).

Although the lower adenosine levels found in HD models suggest that increasing adenosine could have a protective role in HD, some reports suggest that the adenosine effect could be symptom-specific. Indeed, activation of A2AR has a beneficial effect on the motor impairment, but inactivation of A2AR in some models improves cognitive function (Blum et al., 2018). ADK and ADA contribute to the adenosine tone in the striatum, and some conflicting results obtained in animal models could be due to the specific contribution of these enzymes in compensatory effects among proteins/enzymes that control adenosine homeostasis. For example, ADK transcript and ADK activity are upregulated in the mouse that harbors mutated *HTT* with 187 repeats but not in the one with150 repeats (Kao et al., 2017).

Metformin intake correlates with improved cognitive functions in HD patients suffering from diabetes (Hervas et al., 2017). It is known that metformin has a protective effect in HD cell models through activation of AMPK and modulation of mitochondrial dynamics (Jin et al., 2016). However, nuclear localization of AMPKα1 has been reported in the brain tissue of HD patients and it has been demonstrated to potentiate striatal neurodegeneration in HD models (Ju et al., 2011) (Table 1). This raises the possibility that there could be a therapeutical window for AMPK activation and that it should be used at early stages since activation during the late stages of HD might be deleterious. However, more research is necessary to understand the role of AMPKα1 and the possible protective effects of metformin on targets different from AMPK.

## Amyotrophic Lateral Sclerosis

ALS is characterized by degeneration of both upper and lower motor neurons which leads to progressive weakness and ultimately to death. In addition, there might be extra-motor manifestations including alteration of some cognitive functions, and processing of emotions. In about 10% of cases, frontotemporal dementia has been associated with ALS (Benbrika et al., 2019). Ninety per cent of ALS cases are sporadic and more than 35 genes have been linked to the disease. The main genes involved in the familial forms are those coding for the superoxide dismutase (SOD1), TAR DNA binding protein (TARDBP), chromosome 9 open reading frame 72 (C9orf72) and fused in sarcoma (FUS). Disturbances in RNA metabolism, impaired protein homeostasis, nucleocytoplasmic transport defects, impaired DNA repair, excitotoxicity, mitochondrial dysfunction, oxidative stress, axonal transport disruption, neuroinflammation, oligodendrocyte dysfunction, and vesicular transport defects have been proposed to contribute to ALS pathogenesis (Rothstein, 2009; Mejzini et al., 2019; Floare and Allen, 2020).

It has been hypothesized that, at least in some types of ALS, neurodegeneration could be the result of a higher susceptibility to oxidative stress and experiments designed to test it have pointed to the involvement of AMPK activation. The transgenic mouse SOD1(G93A), that overexpresses a human SOD1 with the substitution of glycine 93 to alanine, is the model of a fifth of the familial cases of ALS (Rosen et al., 1993). Embryonic neural stem cells from these mice exhibited more phosphorylated AMPK and were more susceptible to apoptosis in the presence of oxidative stress compared to those obtained from wild type animals, while treatment with compound C, an inhibitor of AMPKα, attenuated the effects of H_2_O_2_ (Sui et al., 2014). Strong activation of AMPK was also found in lumbar spinal cords of SOD1(G93A) mice (Coughlan et al., 2015). However, when these mice were treated with the AMPK activator latrepirdine from postnatal day 70 to day 120, they showed a delayed symptom onset and a significant increase in life span compared to untreated mice (Coughlan et al., 2015). These protective effects were not observed when stimulation of AMPK was obtained through a calorie-restricted diet. Indeed, this diet reduced motor neuron survival and reduced lifespan, while lifespan was increased and disease onset was delayed in the SOD1(G93A) mice fed with a high fat diet (Zhao et al., 2015). These experiments are summarized in Table 2. Therefore, these results point to inhibition, and not to activation of AMPK as a therapeutic strategy for ALS.

**TABLE 2:** Involvement of adenosine metabolism enzymes and receptors in ALS.

Enzyme/receptor	Treatment	Model	Expression/activity	Effect	References
ADA		Astrocytes from C9orf72 ALS patients	Decreased expression	Increased cocultured motor neuron cytotoxicity	Allen et al. (2019)
AMPK		ALS patient-derived mesenchymal stem cells	Decreased expression		Yun et al. (2019)
AMPK	Resveratrol (activator)	ALS patient-derived mesenchymal stem cells	Increased expression and activation	Increased differentiation	Yun et al. (2019)
AMPK	latrepirdin (activator)	SOD1(G93A) mouse	Increased activity	Delayed disease progression	Coughlan et al. (2015)
AMPK	calorie restricted diet (activator)	SOD1(G93A) mouse	Increased activity	Decreased neuronal survival and lifespan	Zhao et al. (2015)
A2AR		Human ALS lymphocyte	Increased expression		Vincenzi et al. (2013)
A2AR		Human, spinal cord	Increased expression		Ng et al. (2015)
A2AR		SOD1(G93A) mouse, spinal cord	Increased expression (disease onset)		Ng et al. (2015)
A2AR		SOD1(G93A) mouse, spinal cord	Decreased expression (later disease stage)		Potenza et al. (2013)
A2AR	Knockout inhibitor	SOD1(G93A) mouse		Increased neuronal survival, delayed disease progression	Ng et al. (2015)
A2AR	caffeine (non selective antagonist)	SOD1(G93A) mouse		Decreased survival	Potenza et al. (2013)

Although many studies have shown that purinergic signaling is altered in ALS, it is not yet clear whether these changes are compensatory or causative. An up-regulation of A2ARs was observed in lymphocytes of ALS patients with respect to healthy subjects (Vincenzi et al., 2013), and, at the symptomatic onset, in the spinal cords of SOD1(G93A) mice and end-stage human ALS spinal cords (Ng et al., 2015). In addition, adenosine induced embryonic stem cell-derived motor neuron cell death in cultures while pharmacological inhibition and partial genetic ablation of A2AR protected motor neurons from cell death and delayed disease progression of SOD1(G93A) mice (Ng et al., 2015) (Table 2). Sebastiao et al. (2018) have suggested that A2AR stimulation or inhibition could play a different role at early and late stages of ALS, since accumulating lines of evidence indicate a beneficial role of both agonists and antagonists of A2AR.

Recent studies have shown that mesenchymal stem cells derived from ALS patients have limited stem cell capacities and exhibit cellular senescence phenotype suggesting that they should be improved before being used in autologous stem cell therapy. Yun et al. (2019) have reported down-regulated AMPK/sirtuin 1 signaling in these cells and have found that resveratrol, which independently stimulates both AMPK and sirtuin 1 (Dasgupta and Milbrandt, 2007; Gertz et al., 2012) restored AMPK/sirtuin 1 and increased differentiation of mesenchymal stem cells into neuron-like cells.

Another recent interesting observation regarding adenosine metabolism has been reported in ALS characterized by a massive hexanucleotide repeat expansion within *C9orf72* (DeJesus-Hernandez et al., 2011; Renton et al., 2011). ADA levels are reduced in fibroblasts, and in both astrocytes and neurons obtained from induced-neuroprogenitor cells from individuals with either C9orf72 mutation or with sporadic ALS (Allen et al., 2019) (Table 2). *In vitro* experiments have shown that, when ADA reduction was bypassed by inosine supplementation, the bioenergetic flux and ATP levels increased in induced-astrocytes and that the induced astrocyte-mediated motor neuron cell death was reduced (Allen et al., 2019). Allen et al. (2019) have suggested that inosine supplementation, in combination with modulation of the level of ADA may represent a beneficial therapeutic approach in ALS patients.

## Concluding Remarks

Adenosine and ATP can be released by neurons, astrocytes, oligodendrocytes and microglia and these cells express receptors for both signaling molecules. Adenosine not only acts as neurotransmitter and neuromodulator, but it is also a metabolic sensor in the brain and contributes to the communication between the different cells fine-controlling synaptic circuitry and neuroinflammation. Nucleoside transporters and/or enzymatic activities involved in the metabolism of adenosine, by affecting the levels of both ATP and adenosine, and therefore the activity of adenosine and ATP receptors, impact on many physiological processes. They could have a role in the onset or the development of central nervous system disorders, and be possible targets of drugs for their treatment. The adenosine concentration must be strictly regulated. Indeed, adenosine (and deoxyadenosine) accumulation such as in ADA deficiency, results in severe combined immunodeficiency associated with neurological deficits. Decrease of adenosine is observed in several pathological conditions, as in epilepsy, in which ADK is increased. ADK not only decreases adenosine levels, but also regulates the transmethylation pathway. Both increase in adenosine and DNA methylation inhibition have a protective role in this pathology. Some studies point to the use of ENT inhibition to increase adenosine levels and ENT inhibitors with the ability to cross the brain blood barrier have been used with antiepileptic activity in animal models. The use of ENT1 inhibitors has also been proposed for HD. In this regard, most studies would find a sound support from the setting-up of reliable techniques for the direct determination of adenosine levels in different physiological and pathological conditions.

Many times, the information available from cell and animal models and patients does not clarify whether the changes in adenosine metabolism or signaling are causative or compensative. However, it has to be considered that often compensatory changes occur during the development of the diseases, and there could be temporal windows when a drug could be effective. In other cases, when the energetic state of the cells is involved, modulation of AMPK has been hypothesized to be a target to improve neurodegeneration and/or cognitive impairment. Although stimulation of AMPK improves impairments in stem cell properties derived from ALS patients, the studies in mouse models indicate that different effects could result from distinct activation pathways of the enzyme, and from the AMPK isozyme localization. Notably, in diabetic patients with AD or HD comorbidity, metformin appears to prevent cognitive decline. Increased knowledge on adenosine metabolism in diseases could provide new tools of intervention. As an example, in the C9orf72 type of ALS, reduced ADA activity and *in vitro* models of disease have led to the hypothesis that inosine could have a protective effect. However, it is clear that much research is still necessary to understand the adenosine metabolism and the cross-talk of different cell types in different regions of the brain and also their temporal variations under physiological and pathological conditions in order to design new therapeutical tools.

## Glossary

AD: Alzheimer’s disease
ADA: adenosine deaminase
ADF: astrocytoma cell line
ADK: adenosine kinase
ADK_L_: ADK-long
ADK_S_: ADK-short
ALS: amyotrophic lateral sclerosis
AMPD: AMP deaminase
AMPK: AMP-activated protein kinase
C9orf72: chromosome 9 open reading frame 72
CD26: dipeptidyl peptidase-4
CD39: ectonucleoside triphosphate diphosphohydrolase
CD73: ecto-5′- nucleotidase
CNT1-3: concentrative nucleoside transporter 1-3
dATP: deoxyadenosine triphosphate
ENT1-4: equilibrative nucleoside transporters 1–4
FUS: fused in sarcoma
GRP58: glucose-regulated protein 58
GSCs: glioblastoma stem-like cells
HD: Huntington’s disease
HPRT: hypoxanthine-guanine phosphoribosyltransferase
HSP: hereditary spastic paraplegia
HTT: huntingtin
IMP: inosine monophosphate
iPSCs: induced pluripotent stem cells
NBTI: nitrobenzythioinosine
NT5C1: cytosolic 5′-nucleotidase I
NT5C2: cytosolic 5′-nucleotidase II
PD: Parkinson’s disease
PNP: purine nucleoside phosphorylase
PRPP: 5-phosphoribosyl-1-pyrophosphate
SAH: S-adenosyl homocysteine
SAHH: S-adenosyl homocysteine hydrolase
SAM: S-adenosylmethionine
SCID: severe combined immunodeficiency
SOD1: superoxide dismutase 1
TARDBP: TAR DNA binding protein
